# Preliminary Development of a Peer Willingness Scale for Depression and Analysis of Associated Factors Among Chinese College Students: A Cross‐Sectional Study

**DOI:** 10.1155/da/3448143

**Published:** 2026-10-17

**Authors:** Yating Fu, Danfeng Wang, Leyi Xu, Yue Yu, Jianliang Zou, Zhipeng Liu, Xiaoyu Zhang

**Affiliations:** ^1^ School of Public Health Wenzhou Medical University Wenzhou Zhejiang China wmu.edu.cn; ^2^ School of Rehabilitation Medicine Wenzhou Medical University Wenzhou Zhejiang China wmu.edu.cn

**Keywords:** bystander intervention, college students, depression symptoms, peer support

## Abstract

**Background:**

While peer support can alleviate depressive symptoms of college students, peers’ willingness to intervene and factors associated with this willingness remain understudied. This study aims to (1) develop and validate a Bystander Intervention Willingness for Depression (BIWD) scale for Chinese college students and (2) identify key sociodemographic and sociopsychological correlates of willingness.

**Methods:**

A cross‐sectional study was conducted from May to June 2024. A 13‐item BIWD scale based on the Bystander Intervention Model was assessed using exploratory (*n* = 217) and confirmatory (*n* = 1008) factor analyses. Linear regression models examined associations between BIWD scores and sociodemographic factors, social perceptions, and mental health indicators.

**Results:**

The BIWD scale comprised four dimensions: depression‐related cognition, intervention responsibility, bystander effect resistance, and self‐efficacy. Older age, higher socioeconomic status, stronger personal anti‐stigma attitude, greater interpersonal closeness, and stronger social support were associated with higher BIWD scores. Students with depressive or anxiety symptoms showed higher depression‐related cognition, but lower intervention responsibility, self‐efficacy, and higher susceptibility to bystander effects.

**Conclusions:**

Findings support the BIWD scale as a useful tool for assessing peer intervention willingness. Initiatives to reduce personal stigma, family functioning enhancement, and targeted self‐efficacy training, especially for students experiencing mental health symptoms, may strengthen peer‐based support for depression.

## 1. Introduction

Depression is a common mental health disorder, affecting ~280 million people worldwide [1]. The global burden extends to specific subpopulations, notably college students, who are navigating the challenging transition from academia to society. During this period, various factors exacerbate their psychological distress, including parental expectations, academic pressure, employment uncertainty, and interpersonal interactions [2, 3]. A meta‐analysis of 100,187 college students showed that the global prevalence of depressive symptoms was 33.6%, which was significantly higher than the average prevalence of depressive symptoms in the general population [4, 5]. Therefore, it is important to identify early signs of depression in college students so as to facilitate timely intervention and treatment.

A large number of research on mental health initiatives has emphasized the role of peer support in the early identification and treatment of depression in adolescents and young adults [6]. Nevertheless, most existing studies have focused on peer support provided by trained individuals (e.g., peer mentors). This kind of support typically requires students with depression to take the initiative to seek help—an action they are often reluctant to take due to stigma, limited trust, and poor mental health literacy [7, 8]. In contrast, during the early or mild stages of depression, ordinary peers who spend substantial time with college students are often better positioned to notice subtle signs and offer informal support. Yet, few studies have explored the willingness of ordinary college students to support their depressed peers and the factors affecting such willingness.

Unfortunately, informal support networks often lack the knowledge and skills to intervene [9]. College students may face various challenges when attempting to support peers with depression, such as difficulty in recognizing depression signals, concerns about the consequences of intervention, and a lack of confidence in their ability to provide effective support. To better motivate and equip peers for intervention, the Bystander Intervention Model may be helpful. Originally proposed by Darley and Latané in 1968 to explain bystander responses to emergencies [10], the model has subsequently been extended to potentially harmful or problematic situations, including mental health concerns [11]. It comprises five sequential steps: (1) noticing something potentially concerning is happening; (2) interpreting the situation as potentially harmful or an emergency; (3) assuming responsibility to intervene; (4) knowing how to intervene; and (5) taking action [11]. When applied to depression, the model does not construe depression itself as an acute emergency; rather, it describes how peers notice potentially concerning signs, recognize that these signs may warrant attention or support, assume responsibility, determine an appropriate response, and take supportive action.

A cross‐sectional study in Singapore using the Bystander Intervention Model developed a preliminary tool to assess the likelihood of intervention and found that older age, past experience with mental health, and having family members or friends with a mental illness were associated with the likelihood of bystander intervention [12]. Notably, this study also found that non‐Chinese college students were more likely to take responsibility for helping a depressed peer than their Chinese counterparts. These findings highlight the influence of the cultural context and underscore the necessity of developing culturally adapted assessment tools. In light of this, the present study aims to (1) develop a preliminary scale, based on the Bystander Intervention Model, to quantify Chinese college students’ willingness to intervene with peers experiencing depression and (2) identify the sociodemographic and sociopsychological factors associated with such willingness, thereby informing future peer‐based mental health interventions.

## 2. Methods

### 2.1. Data Collection Procedure

This cross‐sectional study was conducted from May to June 2024 in Wenzhou, Zhejiang Province, China. Only undergraduate students aged 18–25 years were eligible for participation, while uncooperative individuals and those providing invalid responses (determined by abnormal response times, consecutive identical answers, or logical inconsistencies in related questions) were excluded.

A pilot study was first conducted at Wenzhou Medical University to preliminarily refine the self‐developed questionnaire. Of the 235 questionnaires collected, 217 valid responses were retained for analysis after rigorous screening. For the formal study, a two‐stage sampling method was employed. In the first stage, the 11 eligible institutions were stratified by institution type into universities (*n* = 3) and colleges (*n* = 8), from which 2 universities and 3 colleges were randomly selected from the respective strata. In the second stage, convenience sampling was used to invite students through face‐to‐face interactions. Efforts were made to enhance sample representativeness by selecting high‐traffic locations (e.g., dormitories and cafeterias) to capture a diverse range of participants. Participants who agreed to participate in the study provided informed consent and completed the survey by scanning the QR codes linked to the online questionnaires hosted on the Questionnaire Star platform (https://www.wjx.cn/). A total of 1542 questionnaires were distributed, and 1008 valid responses were obtained, yielding a response rate of 65.37%.

### 2.2. Ethical Consideration

Ethics approval was obtained from the Institutional Review Board of Wenzhou Medical University (Number 2024030). The study was conducted in accordance with the Declaration of Helsinki and relevant institutional ethical guidelines. Participation was voluntary, and informed consent was obtained from all participants.

### 2.3. Measures

#### 2.3.1. Sociodemographic Characteristics

The sociodemographic characteristics included age, sex (male/female), ethnicity (Han/others), perceived family economic status (poor/average/good), major (liberal arts/nonliberal arts), grade (lower‐grade: years 1–2; upper‐grade: years 3–4+), only‐child status (no/yes), single‐parent family (no/yes), parental education (low: ≤9 years of compulsory education; high: ≥high school), and parenting style (democratic/nondemocratic).

#### 2.3.2. Social Perceptions

Social perceptions were assessed across three dimensions: (1) interpersonal closeness, (2) personal stigma toward mental illness, and (3) perceived social support. Interpersonal closeness was assessed using the single‐item pictorial Inclusion of Other in Self (IOS) Scale [13], which presents seven pairs of circles with different degrees of overlap (1 = no overlap; 7 = full overlap), with higher scores indicating greater closeness. Personal stigma toward mental illness was assessed using three items adapted from Eisenberg et al. [14]. The scale comprised two positively worded items (“I would not mind being close friends with someone with a history of mental illness”; “I believe someone with a history of mental illness to be as trustworthy as anyone else”) and one reverse‐scored item (“I would not think highly of someone with a history of mental illness”). Each item was rated on a 5‐point Likert scale (1 = strongly disagree to 5 = strongly agree). After reverse scoring the third item, total scores ranged from 3 to 15, with higher scores indicating stronger personal anti‐stigma attitudes toward mental illness (i.e., lower personal stigma). Perceived social support was assessed using three items adapted from a previously used measure of classmate support [15]. The items measured three domains: (1) emotional support (“I can receive adequate emotional support from family/friends/teachers when needed”), (2) instrumental support (“I can obtain practical help from others when facing difficulties”), and (3) esteem support (“I receive positive feedback that enhances my self‐worth”). Each item was rated on a 5‐point Likert scale, with total scores ranging from 3 to 15; higher scores indicated stronger perceived support.

#### 2.3.3. Mental Health

College students’ mental health status was assessed using four validated instruments: (1) subjective happiness: measured using the 4‐item Subjective Happiness Scale (SHS) on a 7‐point Likert scale [16], with higher scores indicating greater happiness (total range: 4–28; Cronbach’s *α* = 0.82); (2) anxiety: evaluated through the 2‐item Generalized Anxiety Disorder scale (GAD‐2) [17], with higher scores indicating greater anxiety (total range: 0–6; *α* = 0.76); (3) depressive symptoms: assessed via the 9‐item Patient Health Questionnaire (PHQ‐9) [18], with higher scores denoting more severe depressive symptoms (total range: 0–27; *α* = 0.89); (4) perceived stress: measured by the 4‐item Perceived Stress Scale (PSS‐4) [19], which includes two positively worded (reverse‐scored) and two negatively worded items, rated on a 5‐point Likert scale. Higher scores indicated greater perceived stress (total range: 0–16; *α* = 0.85).

#### 2.3.4. Bystander Intervention Willingness

The Bystander Intervention Willingness for Depression (BIWD) scale was grounded in Latané and Darley’s five‐stage Bystander Intervention Model (noticing, interpretation, responsibility assumption, knowledge acquisition, and implementation). The items were designed to assess students’ willingness to provide peer support at each step of the bystander intervention process in the context of depression. The 13‐item scale comprised four dimensions: (1) depression‐related cognition, (2) intervention responsibility, (3) bystander effect resistance, and (4) self‐efficacy. Items in the “bystander effect resistance” dimension were reverse‐scored to ensure that higher scores consistently reflected lower susceptibility to the bystander effect. All items were rated on a 5‐point Likert scale. Total BIWD scores ranged from 13 to 65, with higher total scores indicating a greater willingness to intervene.

### 2.4. Statistical Analysis

Continuous variables were summarized as the mean and standard deviation (SD), while categorical variables were reported as frequencies and percentages. To establish the construct validity of the BIWD scale, both exploratory factor analysis (EFA) and confirmatory factor analysis (CFA) were conducted. EFA was performed on 217 pilot responses using principal component analysis (PCA) with varimax rotation in SPSS (version 26.0). The Kaiser–Meyer–Olkin (KMO) measure was used to verify sampling adequacy (threshold: >0.6), and Bartlett’s test of sphericity was conducted to verify factorability (significant at *p* < 0.05). CFA was then applied to the full sample (*N* = 1008) using maximum likelihood estimation in AMOS 26.0. Model‐fit indices, including *χ* ^2^, df, *χ* ^2^/df, RMSEA, SRMR, CFI, and TLI, were calculated in R version 4.5.2 using the lavaan package (version 0.7–2). Values of *χ* ^2^/df < 3, RMSEA < 0.08, SRMR < 0.10, and CFI and TLI > 0.90 were considered indicative of acceptable fit, whereas values of *χ* ^2^/df < 2, RMSEA < 0.05, SRMR < 0.05, and CFI and TLI > 0.97 were considered indicative of good fit [20, 21]. Because multivariate normality was violated, the Bollen‐Stine bootstrap procedure with 2000 bootstrap samples was additionally conducted [22, 23], and the bootstrap‐derived fit indices were summarized descriptively [24]. Linear regression models were used to assess associations of willingness to intervene, reflected as total BIWD scores, with sociodemographic characteristics, social perceptions, and mental health indicators in R (version 4.5.2). Age and sex were retained a priori, and other sociodemographic variables with *p* < 0.05 in the significance test were selected for inclusion in the multivariate linear regression model. Statistical significance was set at *p* < 0.05 (two‐tailed).

## 3. Results

### 3.1. Sociodemographic Characteristics

The sociodemographic characteristics of the participants are presented in Table 1. The majority of respondents were female (59.69%), of Han ethnicity (97.12%), perceived their family economic status as average (65.77%), and came from two‐parent households (91.47%). Additionally, about three‐quarters perceived their parents’ parenting style as democratic. Descriptive statistics for the social perception measures and mental health outcomes are presented in Table 1.

**Table 1:** Sociodemographic and sociopsychological factors of participants.

Variable	Participants (*n* = 1008)
Age^a^, year	20.94 ± 1.45
Sex	
Male, *n* (%)	406 (40.31)
Female, *n* (%)	602 (59.69)
Ethnicity	
Han, *n* (%)	979 (97.12)
Others, *n* (%)	29 (2.88)
Major	
Liberal arts, *n* (%)	497 (49.31)
Nonliberal arts, *n* (%)	511 (50.69)
Grade	
Lower‐grade, *n* (%)	575 (57.04)
Higher‐grade, *n* (%)	433 (42.96)
Family economic status	
Poor, *n* (%)	145 (14.39)
Average, *n* (%)	663 (65.77)
Good, *n* (%)	200 (19.84)
Only‐child status	
No, *n* (%)	566 (56.15)
Yes, *n* (%)	442 (43.85)
Single‐parent household	
No, *n* (%)	922 (91.47)
Yes, *n* (%)	86 (8.53)
Paternal education level	
Low‐education, *n* (%)	498 (49.40)
High‐education, *n* (%)	510 (50.60)
Maternal education level	
Low‐education, *n* (%)	549 (54.46)
High‐education, *n* (%)	459 (45.54)
Parenting style	
Democratic, *n* (%)	742 (73.61)
Nondemocratic, *n* (%)	266 (26.39)
Personal anti‐stigma attitude score^a^, range 3–15	11.42 ± 2.02
Perceived social support^a^, range 3–15	12.11 ± 2.06
Interpersonal closeness^a^, range 1–7	3.50 ± 1.49
Subjective happiness^a^, range 4–28	19.00 ± 3.87
Depressive symptoms^a^, range 0–27	6.57 ± 2.98
Anxiety^a^, range 0–6	2.09 ± 1.43
Perceived stress^a^, range 0–16	7.41 ± 5.33

^a^Continuous variables were presented as mean ± SD.

### 3.2. Reliability and Validity Assessment of the Bystander Intervention Scale

#### 3.2.1. EFA

EFA was conducted using data from the pilot study (*n* = 217). The KMO and Bartlett’s sphericity test (Table S1) confirmed the adequacy of the sample and the suitability of the data for factor analysis. PCA with varimax rotation was employed to explore the factor structure. Items with factor loadings below 0.40 or cross‐loadings were excluded. As shown in Table S2, four factors with eigenvalues greater than 1 were identified, accounting for 65.226% of the total variance. The four factors were labeled as follows: (1) depression‐related cognition (items 1 and 2); (2) intervention responsibility (items 4, 8, 9, and 10); (3) bystander effect resistance (items 11, 12, and 13); and (4) self‐efficacy (items 3, 5, 6, and 7). The items in each factor are displayed in Table 2.

**Table 2:** Rotated factor matrix from the principal component analysis.

Items		Loading	Cronbach’s *α*
Factor 1: depression‐related cognition		—	0.699
I1	I know that some students in my university have symptoms of depression	0.844	—
I2	I have seen students in my university show signs of depression	0.846	—
Factor 2: intervention responsibility		—	0.768
I4	I feel that I have a responsibility to help a peer with symptoms of depression	0.641	—
I8	I think my helping behavior can have a positive impact on a peer with depression	0.769	—
I9	I think helping a depressed peer will bring me positive feedback (such as happiness, joy, or a sense of accomplishment, etc.)	0.792	—
I10	If a peer shows signs of depression but I do not provide help, it may have serious consequences	0.636	—
Factor 3: bystander effect resistance (reverse‐scored)		—	0.680
I11	If I know that others are willing to help a depressed peer, my willingness to help will be weakened	0.810	—
I12	I think other people are more capable of helping peers with signs of depression	0.840	—
I13	I do not think I can effectively help a peer with symptoms of depression	0.646	—
Factor 4: self‐efficacy		—	0.804
I3	I have some understanding of depression symptoms and its treatment	0.544	—
I5	I can recognize the signs of depression in my peers in time	0.861	—
I6	I can choose the right time to provide help to peers who show signs of depression	0.774	—
I7	I know how to comfort a peer who is currently suffering from depression	0.830	—

#### 3.2.2. CFA

CFA was conducted using data from the formal study to validate the four main factors identified in the EFA (Figure 1; Table S3). RMSEA (0.076), SRMR (0.081), and CFI (0.919) met the criteria for acceptable fit, whereas TLI (0.893) approached the 0.90 criterion. However, *χ* ^2^/df was elevated (*χ* ^2^(59) = 398.890, *χ* ^2^/df = 6.761), potentially reflecting, in part, the sensitivity of chi‐square‐based indices to large sample sizes (*N* = 1008). The Bollen‐Stine bootstrap test of exact fit remained significant (*p* < 0.001). Following the Bollen‐Stine procedure, the bootstrap‐derived descriptive estimates fell within good‐fit ranges (*χ* ^2^/df = 1.322, RMSEA = 0.018, CFI = 0.995, and TLI = 0.994). Convergent validity (Table S4) was supported by adequate standardized factor loadings ranging from 0.530 to 0.858, squared multiple correlations (SMC: 0.281–0.736), composite reliability (CR: 0.695–0.808), and average variance extracted (AVE: 0.437–0.571). Discriminant validity was established through heterotrait–monotrait ratio (HTMT) values, all of which were below 0.90 (Table S5). Additionally, internal consistency was acceptable, with an overall Cronbach’s *α* of 0.795 and all dimension‐specific α values exceeding 0.65 (Table S6).

**Figure 1:**
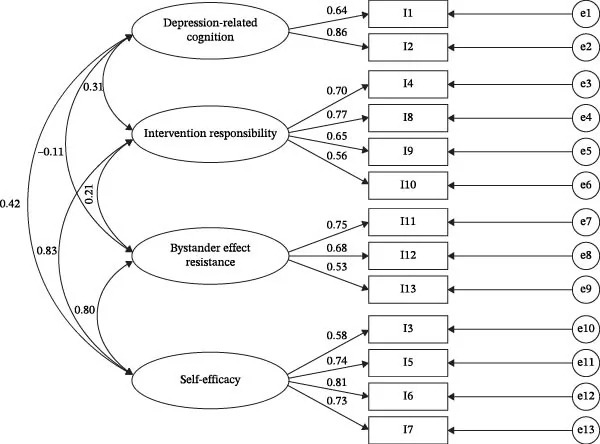
Four‐factor model of Bystander Intervention Willingness for Depression with standardized estimates.

### 3.3. Associations Between Sociodemographic and Sociopsychological Factors and Bystander Intervention Willingness

Table 3 shows that older age, being an only child, higher family economic status, and higher parental education levels were associated with greater willingness to intervene (*p* < 0.05). In contrast, college students who identified as ethnic minorities, majored in nonliberal arts disciplines, came from single‐parent households, or reported nondemocratic parenting styles showed lower willingness to intervene (*p* < 0.05).

**Table 3:** Association between sociodemographic factors and bystander intervention willingness.

Variables	*β* (95% CI)	*p*‐Value
Age	0.275 (0.001, 0.548)	0.049
Sex		
Male	Ref	—
Female	−0.445 (−1.255, 0.366)	0.282
Ethnicity		
Han	Ref	—
Others	−2.833 (−5.206, −0.460)	0.019
Major		
Liberal arts	Ref	—
Nonliberal arts	−0.877 (−1.670, −0.083)	0.030
Grade		
Lower‐grade	Ref	—
Higher‐grade	0.254 (−0.549, 1.057)	0.535
Family economic status		
Poor	Ref	—
Average	2.769 (1.665, 3.873)	<0.001
Good	6.532 (5.219, 7.845)	<0.001
Only‐child status		
No	Ref	—
Yes	1.491 (0.695, 2.287)	<0.001
Single‐parent family		
No	Ref	—
Yes	−1.812 (−3.232, −0.393)	0.012
Paternal education level		
Low‐education	Ref	—
High‐education	2.242 (1.458, 3.025)	<0.001
Maternal education level		
Low‐education	Ref	—
High‐education	1.998 (1.210, 2.787)	<0.001
Parenting style		
Democratic	Ref	—
Nondemocratic	−2.965 (−3.848, −2.081)	<0.001

*Note*: Linear regression model was used.

Table 4 shows that higher intervention willingness was associated with higher personal anti‐stigma attitude scores (*β* = 1.451, 95% CI [1.276, 1.627]), higher levels of perceived social support (*β* = 1.318, 95% CI [1.143, 1.493]), and greater interpersonal closeness (*β* = 1.609, 95% CI [1.360, 1.857]). The associations remained significant after adjusting for sociodemographic factors (*p* < 0.001). Additionally, subjective happiness was positively associated with intervention willingness (*β* = 0.593, 95% CI [0.497, 0.689]), while perceived stress (*β* = −0.824, 95% CI [−0.947, −0.701]), anxiety (*β* = −1.089, 95% CI [−1.359, −0.819]), and depressive symptoms (*β* = −0.270, 95% CI [−0.343, −0.197]) were negatively associated with intervention willingness in Model 1. These associations remained significant after the adjustment.

**Table 4:** Association between sociopsychological factors and bystander intervention willingness.

Variables	Model 1		Model 2	
	*β* (95% CI)	*p*‐Value	*β* (95% CI)	*p*‐Value
Personal anti‐stigma attitude score	1.451 (1.276, 1.627)	<0.001	1.356 (1.189, 1.523)	<0.001
Perceived social support	1.318 (1.143, 1.493)	<0.001	1.138 (0.954, 1.322)	<0.001
Interpersonal closeness	1.609 (1.360, 1.857)	<0.001	1.309 (1.050, 1.567)	<0.001
Subjective happiness	0.593 (0.497, 0.689)	<0.001	0.450 (0.347, 0.552)	<0.001
Perceived stress	−0.824 (−0.947, −0.701)	<0.001	−0.650 (−0.780, −0.519)	<0.001
Anxiety	−1.089 (−1.359, −0.819)	<0.001	−0.752 (−1.023, −0.482)	<0.001
Depressive symptoms	−0.270 (−0.343, −0.197)	<0.001	−0.191 (−0.264, −0.117)	<0.001

*Note*: Linear regression model was used. Model 2 adjusted for age, sex, ethnicity, major, family economic status, only‐child status, single‐parent family, parental education levels and parenting style.

### 3.4. Sensitivity Analyses

Further analyses examined the associations of sociodemographic and sociopsychological factors with the four subdimensions of the BIWD. Detailed associations are presented in Tables S7 and S8. The patterns of associations were largely consistent with those observed for the overall BIWD scores. Older students showed a significant positive association with greater depression‐related cognition (*β* = 0.166, 95% CI [0.094, 0.238]), but their associations with intervention responsibility, bystander effect resistance, and self‐efficacy were not significant. Although no difference was observed in overall BIWD scores by sex, females scored higher on bystander effect resistance (*β* = 0.305, 95% CI [0.006, 0.603]) but lower on intervention responsibility (*β* = −0.313, 95% CI [−0.627, 0.002], *p* = 0.051) and self‐efficacy (*β* = −0.428, 95% CI [−0.795, −0.061]). Exceptions were also noted in relation to anxiety and depressive symptoms. Specifically, higher levels of anxiety (*β* = 0.100, 95% CI [0.027, 0.174]) and depressive symptoms (*β* = 0.038, 95% CI [0.019, 0.058]) were positively associated with greater depression‐related cognition but were negatively associated with scores on the other three dimensions.

## 4. Discussion

This study developed a BIWD scale tailored to Chinese college students to assess their willingness to engage in bystander intervention for peers with depression. Based on a literature review and EFA (*n* = 217), four key dimensions were identified: (1) depression‐related cognition, (2) intervention responsibility, (3) bystander effect resistance, and (4) self‐efficacy. The dimension of “depression‐related cognition” aligns with the “awareness of depression among peers” factor in the existing Bystander Intervention Scale for Depression, originally developed for Singapore university students [12]. This dimension reflects an individual’s ability to recognize potentially concerning depressive signs and corresponds to the first two stages of the adapted model: noticing these signs and recognizing that they may warrant attention or support. The “intervention responsibility” dimension pertains to one’s perceived obligation to assist depressed peers and their expectations of positive outcomes, corresponding to the third stage of the model, which involves determining the degree of personal responsibility. “Self‐efficacy” measures confidence in one’s ability to recognize problems and carry out an intervention, corresponding to the final two stages, which involve determining the specific intervention method and implementing the intervention. Given that the bystander effect (a phenomenon in which individuals are less likely to offer help in the presence of others due to a diffusion of responsibility) may hinder intervention [10], a reverse‐scored dimension—“bystander effect resistance”—was included to assess participants’ resistance to this effect, with higher scores reflecting reduced bystander influence and greater willingness to intervene. The validity and reliability of the formal questionnaire were verified by the CFA (*n* = 1008). The model had good convergent validity in terms of structure and good discriminant validity between dimensions.

Linear regression analyses revealed the associations of sociodemographic and sociopsychological factors with Chinese college students’ willingness to intervene when encountering peers with depression. Despite the narrow age range of participants, older students exhibited greater willingness to intervene and higher awareness of depression. A study in Singapore also reported that older students showed higher vigilance toward possible symptoms of depression and greater knowledge of appropriate intervention strategies [12]. Older students typically have access to a wider array of information channels, which enhances their capacity to acquire relevant knowledge and thereby increases their likelihood of implementing interventions compared to that of younger students. In addition, females showed greater resistance to the bystander effect than males, potentially attributed to their greater prosocial and empathetic traits [25]. However, we also noticed that females exhibited lower self‐efficacy compared to males, which could be explained by their tendency toward greater honesty and humility [26], potentially resulting in more conservative self‐assessments of their abilities and confidence. This differential association with subdimensions could explain the lack of significant gender differences in overall BIWD scores. Family factors (e.g., higher perceived family economic status, two‐parent households, higher parental education, and a democratic parenting style) were positively associated with intervention willingness. These associations may be mediated through enhanced family functioning (e.g., emotional support and healthy interpersonal relationships), which has been linked to greater prosocial and altruistic behavior [27].

As for the mental health status, the positive association between subjective happiness and intervention willingness observed in our study is consistent with the evidence that happiness promotes prosocial behavior [28]. In addition, the willingness to intervene was negatively associated with perceived stress, anxiety, and depressive symptoms. Similarly, research conducted in Sri Lanka observed that individuals with experience of depression demonstrated reduced willingness to provide assistance to depressed peers [29]. A meta‐analysis showed that better mental health was associated with a higher score of extraversion among Chinese individuals [30], suggesting that college students experiencing high levels of stress, anxiety, and depressive symptoms may exhibit reduced social proactivity, thereby diminishing their intervention willingness.

Notably, we found that college students with higher levels of depressive symptoms and anxiety had higher scores on “depression‐related cognition” but lower scores on “intervention responsibility,” “bystander effect resistance,” and “self‐efficacy.” While prior research has shown a negative association between depressive symptoms and depression literacy [31], the findings of the present study suggested the opposite pattern. This divergence may stem from the specific characteristics of the college student population surveyed. Within the university environment, there are typically greater opportunities for participation in mental health support programs or psychological counseling. Consequently, individuals with higher levels of depressive symptoms may possess more extensive knowledge about depression and related concepts. However, despite high awareness of depression, such students frequently exhibit lower levels of prosocial behavior [32], displaying a reduced willingness to assist others, particularly regarding intervention with peers experiencing depression. The finding highlights the importance of targeted interventions aimed at enhancing self‐efficacy and perceived responsibility among students experiencing mental health symptoms to promote active peer support.

Our study found that a higher personal anti‐stigma attitude score was positively associated with the overall BIWD scores and all four dimensions, highlighting the role of anti‐stigmatization in facilitating peer support [33]. Consistent with previous research, stigmatization of mental illness tends to undermine college students’ intention to help depressed peers or provide Mental Health First Aid (MHFA) [29, 34]. Similarly, studies among adolescents have also shown a negative association between personal stigma toward mental illness and the intention to assist depressed peers [35]. These findings underscore the importance of reducing personal stigma toward mental illness as a critical intervention target, not only to foster a supportive campus environment but also to enhance the effectiveness of peer‐led mental health interventions.

Our study also found that higher levels of interpersonal closeness and perceived social support were associated with an increased willingness to intervene. Studies conducted in Australia and Sri Lanka have also identified social distance as a significant negative factor influencing peer‐helping behaviors toward individuals with suspected mental disorders in youth populations [29, 35]. On the other hand, social support fosters the development of altruistic behavior in adolescents, enhancing their confidence in navigating depression‐related challenges and empowering them to support their peers’ recovery‐oriented actions [27].

This study has certain limitations. First, the sample was drawn exclusively from a single region, which may limit the generalizability of our findings. Second, the use of convenience sampling may introduce a volunteer bias. Finally, although adjustments were made for multiple variables, the possibility of residual confounding cannot be fully excluded.

## 5. Conclusions

This study extends the application of the Bystander Intervention Model to peer support for depression by assessing college students’ willingness to intervene and identifying associated sociodemographic and sociopsychological factors, thereby providing valuable insights into the development of targeted peer‐support interventions. However, more studies with large‐scale, multicenter designs are needed to further identify and validate the factors affecting college students’ intervention behaviors toward peers with depression.

**Nomenclature**
BIWD: Bystander Intervention Willingness for Depression
IOS: Inclusion of Other in Self
SHS: Subjective Happiness Scale
GAD‐2: The 2‐item Generalized Anxiety Disorder scale
PHQ‐9: The 9‐item Patient Health Questionnaire
PSS‐4: The 4‐item Perceived Stress Scale
SD: Standard deviation
EFA: Exploratory factor analysis
CFA: Confirmatory factor analysis
PCA: Principal component analysis
KMO: Kaiser–Meyer–Olkin
RMSEA: Root mean square error of approximation
SRMR: Standardized root mean square residual
CFI: Comparative fit index
TLI: Tucker–Lewis index
SMC: Squared multiple correlation
AVE: Average variance extracted
CR: Composite reliability
HTMT: Heterotrait–monotrait ratio
MHFA: Mental Health First Aid.

## Author Contributions

Yating Fu and Danfeng Wang curated and analyzed the data, conducted the investigation, and contributed to both the original draft and manuscript revisions. Leyi Xu participated in the investigation, contributed to data interpretation and visualization, and assisted in manuscript revision. Yue Yu and Jianliang Zou contributed to the investigation and manuscript revision. Zhipeng Liu contributed to the study’s conceptualization, methodology, validation, funding acquisition, and participated in manuscript revision. Xiaoyu Zhang was responsible for conceptualization, methodology, project administration, supervision, funding acquisition, and contributed to manuscript revision.

## Funding

This study was supported by the Natural Science Foundation of Zhejiang Province (Grants LQ23H260003 and LQN25H260004).

## Disclosure

All authors reviewed and approved the final manuscript.

## Ethics Statement

This study was conducted in strict compliance with the ethical principles outlined in the Declaration of Helsinki and received formal approval from the Institutional Review Board (IRB) of Wenzhou Medical University (Approval Number 2024030) prior to study implementation. All participants were informed of the study’s purpose, procedures, potential risks, and benefits, and they consented to participate.

## Consent

The authors have nothing to report.

## Conflicts of Interest

The authors declare no conflicts of interest.

## Supporting Information

Additional supporting information can be found online in the Supporting Information section.

## Supporting information


**Supporting Information** Supporting Information associated with this article includes Tables S1–S8. These tables present the KMO and Bartlett’s test results (Table S1), principal component analysis results (Table S2), observed and bootstrap‐derived model fit indices (Table S3), convergent validity, discriminant validity, and reliability results (Tables S4–S6), and dimension‐specific regression analyses (Tables S7 and S8).

## Data Availability

The datasets used and/or analyzed during the current study are available from the corresponding author upon reasonable request.
