# Supplementary material for: Preliminary Development of a Peer Willingness Scale for Depression and Analysis of Associated Factors Among Chinese College Students: A Cross‐Sectional Study
Source: Depress Anxiety. 2026 Oct 17;2026:3448143. doi: 10.1155/da/3448143 (PMC13675836; doi:10.1155/da/3448143)
Supplement: Supplementary file 1 [file DA-2026-3448143-s001.docx]

**Table S1. KMO and Bartlett's Test**

| Kaiser-Meyer-Olkin Measure of Sampling Adequacy | | 0.798 |
| --- | --- | --- |
| Bartlett's Test of Sphericity | Approx. Chi-Square | 918.290 |
|  | DF | 78 |
|  | Sig. | <0.001*** |

*** *p* < 0.001.

**Table S2. Principal Component Analysis on BIWD**

| Comp. | Initial Eigenvalues | | | Extraction Sums of Squared Loadings | | | Rotation Sums of Squared Loadings | | |
| --- | --- | --- | --- | --- | --- | --- | --- | --- | --- |
|  | Total | % of  Variance | Cumulative % | Total | % of Variance | Cumulative % | Total | % of Variance | Cumulative % |
| 1 | 4.210 | 32.384 | 32.384 | 4.210 | 32.384 | 32.384 | 2.592 | 19.939 | 19.939 |
| 2 | 1.893 | 14.564 | 46.947 | 1.893 | 14.564 | 46.947 | 2.400 | 18.461 | 38.399 |
| 3 | 1.250 | 9.614 | 56.562 | 1.250 | 9.614 | 56.562 | 1.905 | 14.657 | 53.057 |
| 4 | 1.126 | 8.665 | 65.226 | 1.126 | 8.665 | 65.226 | 1.582 | 12.169 | 65.226 |
| 5 | 0.834 | 6.415 | 71.641 |  |  |  |  |  |  |
| 6 | 0.731 | 5.625 | 77.266 |  |  |  |  |  |  |
| 7 | 0.618 | 4.756 | 82.022 |  |  |  |  |  |  |
| 8 | 0.518 | 3.982 | 86.004 |  |  |  |  |  |  |
| 9 | 0.489 | 3.763 | 89.767 |  |  |  |  |  |  |
| 10 | 0.383 | 2.947 | 92.714 |  |  |  |  |  |  |
| 11 | 0.345 | 2.652 | 95.365 |  |  |  |  |  |  |
| 12 | 0.306 | 2.353 | 97.718 |  |  |  |  |  |  |
| 13 | 0.297 | 2.282 | 100.000 |  |  |  |  |  |  |

**Table S3. Observed model fit indices and bootstrap-derived descriptive estimates**

| Fit indices | Acceptable ^a^ | Good fit ^a^ | Observed ML estimate | Bootstrap-derived estimate ^b^ |
| --- | --- | --- | --- | --- |
| χ²/df | <3 | <2 | 6.761 | 1.322 |
| TLI | >0.90 | >0.97 | 0.893 | 0.994 |
| CFI | >0.90 | >0.97 | 0.919 | 0.995 |
| RMSEA | <0.08 | <0.05 | 0.076 ^c^ | 0.018 ^d^ |
| SRMR | <0.10 | <0.05 | 0.081 | N/A |

χ²/df, chi-square divided by degrees of freedom; RMSEA, Root mean square error of approximation; SRMR, Standardized root mean square residual; CFI, Comparative fit index; TLI, Tucker–Lewis index; ML, maximum likelihood; N/A, not applicable.

ᵃ Criteria were based on Hu and Bentler (1998) and Schermelleh-Engel et al. (2003).

ᵇ Bootstrap-derived χ²/df, CFI, TLI, and RMSEA were calculated from the mean discrepancy across 2,000 Bollen-Stine bootstrap samples. SRMR was not derived from this calculation.

ᶜ 90% CI for the observed RMSEA: [0.069, 0.083].

ᵈ 90% bootstrap interval for the bootstrap-derived RMSEA: [0.000, 0.028].

**Table S4. Parameter estimates and convergent validity indicators for each dimension**

| Factor | Items | UnEst | S.E. | t-value | *p* | Est | SMC | AVE | CR |
| --- | --- | --- | --- | --- | --- | --- | --- | --- | --- |
| Depression-related cognition | I1 | 1.000 |  |  |  | 0.637 | 0.406 | 0.571 | 0.722 |
|  | I2 | 1.619 | 0.173 | 9.350 | *** | 0.858 | 0.736 |  |  |
| Intervention responsibility | I4 | 1.000 |  |  |  | 0.704 | 0.496 | 0.454 | 0.767 |
|  | I8 | 1.063 | 0.052 | 20.639 | *** | 0.767 | 0.588 |  |  |
|  | I9 | 0.866 | 0.048 | 17.919 | *** | 0.645 | 0.416 |  |  |
|  | I10 | 0.788 | 0.050 | 15.771 | *** | 0.561 | 0.315 |  |  |
| Bystander effect resistance | I11 | 1.000 |  |  |  | 0.749 | 0.561 | 0.437 | 0.695 |
|  | I12 | 0.801 | 0.063 | 12.774 | *** | 0.684 | 0.468 |  |  |
|  | I13 | 0.713 | 0.059 | 12.176 | *** | 0.530 | 0.281 |  |  |
| Self-efficacy | I3 | 1.000 |  |  |  | 0.582 | 0.339 | 0.516 | 0.808 |
|  | I5 | 1.257 | 0.073 | 17.178 | *** | 0.737 | 0.543 |  |  |
|  | I6 | 1.308 | 0.072 | 18.044 | *** | 0.806 | 0.650 |  |  |
|  | I7 | 1.381 | 0.081 | 17.074 | *** | 0.729 | 0.531 |  |  |

*** *p* < 0.001.

UnEst, unstandardized estimate; S.E., standard error; Est, standardized estimate; SMC, squared multiple correlation; AVE, average variance extracted; CR, composite reliability.

**Table S5. Discriminant validity—Heterotrait–Monotrait (HTMT) ratio**

|  | Depression-related cognition | Intervention responsibility | Self-efficacy | Bystander effect resistance |
| --- | --- | --- | --- | --- |
| Depression-related cognition | —— |  |  |  |
| Intervention responsibility | 0.299 | —— |  |  |
| Self-efficacy | 0.126 | 0.282 | —— |  |
| Bystander effect resistance | 0.446 | 0.806 | 0.147 | —— |

**Table S6. Reliability analysis for each dimension**

| Factor | Cronbach’s Alpha of the dimensions | Total Cronbach’s Alpha |
| --- | --- | --- |
| Depression-related cognition | 0.699 | 0.795 |
| Intervention responsibility | 0.768 |  |
| Bystander effect resistance | 0.680 |  |
| Self-efficacy | 0.804 |  |

**Table S7. Association between sociodemographic factors and four sub-dimension scores of BIWD**

| **Variables** | **Depression-related cognition** | | **Intervention responsibility** | | **Bystander effect resistance** | | **Self-efficacy** | |
| --- | --- | --- | --- | --- | --- | --- | --- | --- |
|  | **β (95% CI)** | ***p*-value** | **β (95% CI)** | ***p*-value** | **β (95% CI)** | ***p*-value** | **β (95% CI)** | ***p-*value** |
| Age | 0.166 (0.094, 0.238) | < 0.001 | 0.079 (-0.027, 0.185) | 0.145 | -0.068 (-0.169, 0.033) | 0.189 | 0.097 (-0.027, 0.221) | 0.125 |
| Sex |  |  |  |  |  |  |  |  |
| Male | Ref |  | Ref |  | Ref |  | Ref |  |
| Female | -0.009 (-0.224, 0.207) | 0.938 | -0.313 (-0.627, 0.002) | 0.051 | 0.305 (0.006, 0.603) | 0.046 | -0.428 (-0.795, -0.061) | 0.022 |
| Ethnicity |  |  |  |  |  |  |  |  |
| Han | Ref |  | Ref |  | Ref |  | Ref |  |
| Others | -0.829 (-1.459, -0.199) | 0.010 | -0.703 (-1.627, 0.221) | 0.136 | -0.362 (-1.241, 0.516) | 0.419 | -0.939 (-2.016, 0.138) | 0.087 |
| Major |  |  |  |  |  |  |  |  |
| Liberal arts | Ref |  | Ref |  | Ref |  | Ref |  |
| Non-liberal arts | -0.194 (-0.405, 0.017) | 0.072 | -0.232 (-0.541, 0.077) | 0.141 | -0.352 (-0.645, -0.059) | 0.019 | -0.100 (-0.460, 0.261) | 0.587 |
| Grade |  |  |  |  |  |  |  |  |
| Lower-grade | Ref |  | Ref |  | Ref |  | Ref |  |
| Higher-grade | 0.356 (0.143, 0.568) | 0.001 | 0.104 (-0.209, 0.416) | 0.515 | -0.302 (-0.598, -0.006) | 0.046 | 0.097 (-0.267, 0.461) | 0.602 |
| Family economic status | |  |  |  |  |  |  |  |
| Poor | Ref |  | Ref |  | Ref |  | Ref |  |
| Average | 0.462 (0.160, 0.764) | 0.003 | 0.664 (0.226, 1.102) | 0.003 | 0.519 (0.093, 0.946) | 0.017 | 1.123 (0.628, 1.619) | < 0.001 |
| Good | 1.101 (0.742, 1.46) | < 0.001 | 1.900 (1.379, 2.420) | < 0.001 | 0.400 (-0.108, 0.907) | 0.123 | 3.132 (2.542, 3.721) | < 0.001 |
| Only-child status |  |  |  |  |  |  |  |  |
| No | Ref |  | Ref |  | Ref |  | Ref |  |
| Yes | 0.368 (0.156, 0.58) | 0.001 | 0.383 (0.073, 0.694) | 0.016 | 0.019 (-0.277, 0.315) | 0.897 | 0.720 (0.359, 1.080) | < 0.001 |
| Single-parent family | |  |  |  |  |  |  |  |
| No | Ref |  | Ref |  | Ref |  | Ref |  |
| Yes | -0.509 (-0.886, -0.132) | 0.008 | -0.434 (-0.986, 0.119) | 0.124 | -0.583 (-1.108, -0.059) | 0.029 | -0.286 (-0.932, 0.359) | 0.384 |
| Paternal education level | |  |  |  |  |  |  |  |
| Low-education | Ref |  | Ref |  | Ref |  | Ref |  |
| High-education | 0.481 (0.272, 0.690) | < 0.001 | 0.535 (0.228, 0.843) | 0.001 | 0.064 (-0.230, 0.358) | 0.669 | 1.161 (0.808, 1.515) | < 0.001 |
| Maternal education level | |  |  |  |  |  |  |  |
| Low-education | Ref |  | Ref |  | Ref |  | Ref |  |
| High-education | 0.458 (0.248, 0.669) | < 0.001 | 0.538 (0.230, 0.847) | 0.001 | 0.110 (-0.185, 0.404) | 0.466 | 0.892 (0.534, 1.249) | < 0.001 |
| Parenting style |  |  |  |  |  |  |  |  |
| Democratic | Ref |  | Ref |  | Ref |  | Ref |  |
| Non-democratic | -0.176 (-0.415, 0.064) | 0.150 | -1.075 (-1.419, -0.731) | < 0.001 | -0.615 (-0.946, -0.284) | < 0.001 | -1.098 (-1.502, -0.695) | < 0.001 |

Linear regression models were used.

**Table S8. Association between sociopsychological factors and four sub-dimension scores of BIWD**

| **Variables** | **Depression-related cognition** | | **Intervention responsibility** | | **Bystander effect resistance** | | **Self-efficacy** | |
| --- | --- | --- | --- | --- | --- | --- | --- | --- |
|  | **β (95% CI)** | ***p*-value** | **β (95% CI)** | ***p*-value** | **β (95% CI)** | ***p*-value** | **β (95% CI)** | ***p*-value** |
| **Model 1** |  |  |  |  |  |  |  |  |
| Personal anti-stigma attitude score | 0.118 (0.066, 0.170) | < 0.001 | 0.511 (0.442, 0.581) | < 0.001 | 0.365 (0.295, 0.434) | < 0.001 | 0.457 (0.373, 0.542) | < 0.001 |
| Perceived social support | 0.121 (0.070, 0.172) | < 0.001 | 0.544 (0.477, 0.611) | < 0.001 | 0.128 (0.057, 0.199) | < 0.001 | 0.525 (0.443, 0.606) | < 0.001 |
| Interpersonal closeness | 0.060 (-0.011, 0.131) | 0.098 | 0.548 (0.450, 0.647) | < 0.001 | 0.364 (0.268, 0.461) | < 0.001 | 0.636 (0.521, 0.751) | < 0.001 |
| Subjective happiness | 0.063 (0.036, 0.090) | < 0.001 | 0.243 (0.206, 0.280) | < 0.001 | 0.012 (-0.026, 0.050) | 0.534 | 0.275 (0.231, 0.318) | < 0.001 |
| Perceived stress | -0.042 (-0.077, -0.007) | 0.019 | -0.282 (-0.331, -0.233) | < 0.001 | -0.165 (-0.213, -0.117) | < 0.001 | -0.335 (-0.391, -0.278) | < 0.001 |
| Anxiety | 0.048 (-0.026, 0.122) | 0.203 | -0.382 (-0.487, -0.276) | < 0.001 | -0.359 (-0.459, -0.259) | < 0.001 | -0.396 (-0.520, -0.273) | < 0.001 |
| Depressive symptoms | 0.027 (0.007, 0.046) | 0.009 | -0.103 (-0.131, -0.075) | < 0.001 | -0.112 (-0.139, -0.086) | < 0.001 | -0.081 (-0.114, -0.047) | < 0.001 |
| **Model 2** |  |  |  |  |  |  |  |  |
| Personal anti-stigma attitude score | 0.103 (0.053, 0.154) | < 0.001 | 0.484 (0.416, 0.552) | < 0.001 | 0.357 (0.288, 0.426) | < 0.001 | 0.415 (0.335, 0.496) | < 0.001 |
| Perceived social support | 0.085 (0.034, 0.137) | 0.001 | 0.505 (0.433, 0.577) | < 0.001 | 0.092 (0.016, 0.168) | 0.018 | 0.448 (0.363, 0.532) | < 0.001 |
| Interpersonal closeness | -0.004 (-0.078, 0.069) | 0.906 | 0.448 (0.344, 0.552) | < 0.001 | 0.384 (0.282, 0.486) | < 0.001 | 0.462 (0.344, 0.581) | < 0.001 |
| Subjective happiness | 0.035 (0.007, 0.063) | 0.016 | 0.206 (0.165, 0.246) | < 0.001 | -0.002 (-0.043, 0.039) | 0.922 | 0.211 (0.165, 0.258) | < 0.001 |
| Perceived stress | -0.005 (-0.041, 0.032) | 0.807 | -0.227 (-0.280, -0.175) | < 0.001 | -0.167 (-0.219, -0.115) | < 0.001 | -0.253 (-0.312, -0.194) | < 0.001 |
| Anxiety | 0.100 (0.027, 0.174) | 0.007 | -0.266 (-0.375, -0.158) | < 0.001 | -0.356 (-0.461, -0.252) | < 0.001 | -0.238 (-0.361, -0.115) | < 0.001 |
| Depressive symptoms | 0.038 (0.019, 0.058) | < 0.001 | -0.077 (-0.107, -0.048) | < 0.001 | -0.108 (-0.136, -0.080) | < 0.001 | -0.046 (-0.079, -0.012) | 0.007 |

Linear regression models were used.

Model 1 did not adjust for any covariates; Model 2 adjusted for age and sex a priori, together with other sociodemographic variables significantly associated with the corresponding BIWD dimension.

Model 2 for “Depression-related cognition” dimension adjusted for age, sex, ethnicity, grade, family economic status, only-child status, a single-parent family, parental education levels.

Model 2 for “Intervention responsibility” dimension adjusted for age, sex, family economic status, only-child status, parental education levels and parenting style.

Model 2 for “Bystander effect resistance” dimension adjusted for age, sex, major, grade, family economic status, a single-parent family and parenting style.

Model 2 for “Self-efficacy” dimension adjusted for age, sex, family economic status, only-child status, parental education levels and parenting style.
